# Comparative analysis of plan quality and delivery efficiency: ZAP-X vs. CyberKnife for brain metastases treatment

**DOI:** 10.3389/fonc.2024.1333642

**Published:** 2024-06-12

**Authors:** Ying Niu, Abdul Rashid, Jui-min Lee, Michael Carrasquilla, Dylan R. Conroy, Brian T. Collins, Andrew Satinsky, Keith R. Unger, Dalong Pang

**Affiliations:** Department of Radiation Medicine, Georgetown University Hospital, Washington, DC, United States

**Keywords:** stereotactic radiosurgery, brain metastases, CyberKnife, ZAP-X, plan quality, delivery efficiency

## Abstract

**Purpose/Objectives:**

ZAP-X, a novel and dedicated radiosurgery (SRS) system, has recently emerged, while CyberKnife has solidified its position as a versatile solution for SRS and stereotactic body radiation therapy over the past two decades. This study aims to compare the dosimetric performance and delivery efficiency of ZAP-X and CyberKnife in treating brain metastases of varying target sizes, employing circular collimation.

**Methods and materials:**

Twenty-three patients, encompassing a total of 47 brain metastases, were included in the creation of comparative plans of ZAP-X and CyberKnife for analysis. The comparative plans were generated to achieve identical prescription doses for the targets, while adhering to the same dose constraints for organs at risk (OAR). The prescription isodose percentage was optimized within the range of 97–100% for each plan to ensure effective target-volume coverage. To assess plan quality, indices such as conformity, homogeneity, and gradient (CI, HI, and GI) were computed, along with the reporting of total brain volumes receiving 12Gy and 10Gy. Estimated treatment time and monitor units (MUs) were compared between the two modalities in evaluating delivery efficiency.

**Results:**

Overall, CyberKnife achieved better CI and HI, while ZAP-X exhibited better GI and a smaller irradiated volume for the normal brain. The superiority of CyberKnife’s plan conformity was more pronounced for target size less than 1 cc and greater than 10 cc. Conversely, the advantage of ZAP-X’s plan dose gradient was more notable for target sizes under 10 cc. The homogeneity of ZAP-X plans, employing multiple isocenters, displayed a strong correlation with the target’s shape and the planner’s experience in placing isocenters. Generally, the estimated treatment time was similar between the two modalities, and the delivery efficiency was significantly impacted by the chosen collimation sizes for both modalities.

**Conclusion:**

This study demonstrates that, within the range of target sizes within the patient cohort, plans generated by ZAP-X and CyberKnife exhibit comparable plan quality and delivery efficiency. At present, with the current platform of the two modalities, CyberKnife outperforms ZAP-X in terms of conformity and homogeneity, while ZAP-X tends to produce plans with a more rapid dose falloff.

## 1 Introduction

Brain metastases are the most prevalent cancerous lesions in the brain, with an estimated incidence rate of 20–40% among cancer patients (1–3). Radiotherapy serves as a viable treatment option for metastatic brain tumors, either as a primary solution or in combination with systemic chemotherapy. Historically, whole brain radiotherapy (WBRT) was the standard approach for patients with or without surgery. However, due to concerns regarding toxicity, WBRT is now commonly deferred (4, 5). As a focal technique that minimizes damage to surrounding healthy tissues, stereotactic radiosurgery (SRS) has emerged as a preferred management option for brain metastases of patients (6). Numerous studies have highlighted the advantages of using SRS or adding SRS to WBRT for brain metastases, such as improved local control, comparable survival rates, and reduced cognitive deterioration (7–9). Consequently, there has been a steady increase in the percentage of patients receiving SRS treatment (10, 11).

The advantages of SRS treatment stem from the dosimetric characteristics of its plans, specifically the steep dose gradient and high dose conformity, which enable dose intensification beyond the capabilities of conventional treatments (12–14). SRS is offered as a treatment solution by various radiation modalities, including cobalt-60 based systems like the Gamma Knife (15, 16), as well as systems with specially equipped linear accelerators (Linacs), such as the Varian Edge (Varian, Palo Alto, CA, USA) (17), Brainlab Novalis (Brainlab, Munich, Germany) (18), CyberKnife (Accuray Inc. Sunnyvale, California) (19) and the more recently developed ZAP-X (ZAP Surgical, San Carlos, CA) (20).

CyberKnife, a robotic radiosurgery system, was introduced commercially in the late 1990’s. Its standout advantage lies in its ability to deliver non-coplanar radiation fields with ease and real-time tumor tracking. Initially developed for treating intracranial lesions, CyberKnife has expanded to treat lesions throughout the body, benefiting thousands of patients worldwide (21). The system features a compact 6-MV X-band Linac and a versatile robotic arm equipped with six joints, allowing for both rotational and translational movement of the radiation source (22). The radiation source follows a predetermined path that connects multiple beam entry locations (i.e., nodes) on a virtual spherical surface. This unique configuration allows for radiation crossfire from nodes distributed across solid angles exceeding 2π steradians. Furthermore, the radiation beams can be precisely directed to non-isocentric directions from each node. CyberKnife possesses a source axial distance (SAD) ranging from 65 to 80 cm, with a dose rate of up to 1000 monitor unit per minute (MU/min). It offers a selection of fixed conical cones and variable circular collimators (i.e., IRIS™) with 12 different collimation diameters from 5 to 60 mm. The IRIS collimator is made of two banks of six tungsten segments, each creating a hexagonal aperture to produce a 12-sided field shape to approximate a circle (23). Notably, a recent addition to the system includes Multileaf collimator (MLC) technology (24). Frameless intracranial treatment is facilitated by the system’s imaging guidance, which employs a pair of orthogonal room-mounted kV generators and panels. This imaging guidance provides reconstructed 3D coordinates of the patient’s skull for precise initial setup and real-time tracking during treatment delivery. To enhance precision, a customized mesh face mask is prepared during simulation phase and is subsequently utilized for treatment. Prior to treatment initiation, any deviation in the initial setup is corrected through the controlled movement of robotic couch. Throughout the delivery of treatment, the real-time patient movement is compensated by the movement of robotic arm (25).

ZAP-X is a cutting-edge platform that emerged in the market just a few years ago. Rather than other SRS systems that require a shielded radiation vault, ZAP-X’s standout feature is its self-contained, self-shielded design (26–28). Its primary focus is on the precise treatment of intracranial lesions without compromising versatility. ZAP-X incorporates a 2.7-MV S-band Linac, which is mounted on a gyroscope-like gantry with independent dual rotating axes, centered around a unique common isocenter. Notably, it offers delivery of non-coplanar radiation beams through moving the radiation source on a virtual spherical surface, covering approximately 2π steradians of solid angles. Additionally, ZAP-X features a compact 45 cm SAD and a dose rate of up to 1500 MU/min. It provides circular collimation with eight different diameters, ranging from 4 to 25 mm. The collimator size can be changed automatically during treatment through a novel tungsten wheel collimator (29). For imaging guidance, ZAP-X employs a gantry-mounted kV imaging system, allowing the capturing of images from specified angles to achieve precise initial skull alignment. This system also facilitates continuous image acquisition at predetermined intervals, ensuring seamless rotation of gantry during treatment delivery. Skull offsets are calculated by aligning the captured images with real-time generated Digitally Reconstructed Radiographs (DRRs). Based on these calculated offsets, the isocenter position is accurately corrected in patient’s head using a patient couch equipped with translational movement capabilities. In line with Cyberknife, ZAP-X also utilizes a customized mesh face mask to support patient immobilization.

Both ZAP-X and CyberKnife utilize compact Linac designs and offer non-coplanar radiation delivery with a large range of beam geometry in terms of solid angle, which is crucial for achieving desired optimized plans in intracranial SRS. However, there are notable differences between the two systems. ZAP-X employs an isocentric technique with couch movement, allowing for the delivery of multiple isocenters within a single treatment. In contrast, CykerKnife delivers non-isocentric beams without requiring couch movement during treatment delivery. Both systems employ circular collimation, offering similar collimating sizes for lesions with small and intermediate volumes. However, CyberKnife offers larger collimator sizes to accommodate lesions with large volumes. It is worth mentioning that ZAP-X, with its shorter SAD and lower beam energy, exhibits characteristics that align more closely with Gamma Knife. A study conducted by Georg et al. focused on the peripheral dose fall-off of ZAP-X using various detectors, and the results indicate that the beam characteristics of ZAP-X are more like those of Gamma Knife (30). The differences in machine characteristics between ZAP-X and CyberKnife may potentially impact their plan parameters, which motivates further investigation into the dosimetric comparison between these two modalities. Romanelli et al. conducted a preliminary dosimetric comparison of trigeminal neuralgia plans between ZAP-X and CyberKnife. They found that the two modalities yielded comparable plans for such functional treatment, and also highlighted the potential clinical value of ZAP-X in low dose region (31). Several studies have investigated the dosimetric characteristic of CyberKnife compared with other techniques, such as Gamma Knife and volumetric modulated arc therapy (VMAT) (32–35). However, to the best of our knowledge, no systematic study has been published comparing the dosimetric performance and delivery efficiency of ZAP-X and CyberKnife for brain metastases with varying target sizes. Therefore, our objective is to perform such a comparison, capitalizing on our extensive experience of CyberKnife SRS and more recent implementation of ZAP-X SRS since 2020.

## 2 Materials and methods

### 2.1 Patient cohort

In this study, a cohort of 23 patients who underwent treatment between 2018 and 2021 were selected. Among these patients, 12 received CyberKnife treatment and 11 patients received ZAP-X treatment. The cohort comprised a total of 47 lesions, which were treated using 28 individual treatment plans. Each plan targeted 1 to 6 metastases. Notably, the anatomical locations of the lesions were well distributed within the cohort, as evidenced by **Table 1**. Additionally, **Table 2** provides an overview of the plan distribution concerning the number of lesions and the volume size of targets. The majority of plans (i.e., 89.3%) targeted 1 or 2 lesions. The plan distribution in terms of the sizes of the targeted lesions displayed a well-balanced representation. To facilitate comparison, an alternative ZAP-X/CyberKnife plan was generated for each original CyberKnife/ZAP-X treatment plan, ensuring attainment of the same clinical goal. The median prescription dose across the 28 plans was 24 Gy (i.e., range, 15–30 Gy), administered over 1 to 5 fractions.

**Table 1:** Summary of tumor locations (47 lesions).

		n	n (%)
Frontal	Left	4	8.5%
	Right	7	14.9%
Temporal	Left	4	8.5%
	Right	3	6.4%
Parieto-occipital	Left	6	12.8%
	Right	10	21.3%
Cerebellar	Left	5	10.6%
	Right	6	12.8%
Vermis		2	4.3%

**Table 2:** Statistics summary of 28 treatment Plans.

		No. of Plans	% of Plans
No. of Lesions per plan	1	18	64.3%
	2	7	25.0%
	4 -6	3	10.7%
Total volume of plan	<1 cc	9	32.1%
	[1 cc, 3 cc)	7	25.0%
	[3 cc, 10 cc)	7	25.0%
	>10 cc	5	17.9%
Fraction No. of plan prescription	1	12	42.9%
	3	13	46.4%
	5	3	10.7%

### 2.2 Imaging and target delineation

The planning CT series was obtained with a slice thickness of no more than 1 mm. To aid in contouring, T1-weighted MRI images with or without contrast in 1 mm thickness were fused with the planning CT. Experienced radiation oncologists delineated the gross tumor volume (GTV) as well as the organs at risk (OARs). The target volume for all patients in the study was defined as the planning target volume (PTV), which incorporated setup margins. In this study, PTV was created from GTV using margins of 0 or 1 mm for all selected patients. The prescription dose for all patients was designated to the PTV. Normal brain tissue was defined as the entire brain excluding the PTV.

### 2.3 Treatment planning

ZAP-X planning was performed using the dedicated ZAP-X treatment planning system (referred to as “ZAP-X TPS”). The ZAP-X TPS incorporates a sphere packing scheme with inverse planning (36) and supports both manual and automatic isocenter placement. Considering the compact design of ZAP-X, a simulation is conducted for each determined isocenter position from sphere packing to establish a safety zone for gantry movement, ensuring collision-free delivery based on a conservative patient model with appropriate size and margins. Subsequently, all available beam angles for all isocenter positions are determined, forming a pool for inverse planning. In the inverse planning process, the weights of the beams in the pool are optimized using linear and quadratic programing, based on the planner’s defined constraints. For each isocenter, the optimized beams with non-zero MUs are connected to form a delivery path using the traveling salesman algorithm, minimizing delivery time while avoiding collision. In cases where the target is small and regular in shape, the isocenters are typically placed at the center of targets with suitable collimator sizes. For larger or irregularly shaped targets, multiple isocenters are employed, with each isocenter covering the target partially. These multiple isocenters are typically positioned near the boundary of the target to minimize the overlaps between shots within target, resulting in a desired plan uniformity.

Treatment planning for CyberKnife was performed using the Accuray Precision 2.0 treatment planning system (referred to as “CyberKnife TPS”) on the CyberKnife VSI platform. In this study, all plans were generated using the variable IRIS™ collimator for the range of tumor size in this study. The CyberKnife TPS offers both short and full paths with different numbers of nodes, and for this study, the full path with a larger number of nodes was utilized. The VOLO optimizer, operating under the inverse planning scheme, was employed (37). The CyberKnife TPS allows for manual or automatic selection of initial collimator sizes for optimization. The choice of collimator sizes depends on the target sizes and the planner’s preference for conformity and homogeneity. Planners can choose to activate multiple collimator sizes to maximize dose-profile sculpting capabilities or select a minimal number of collimator sizes to streamline computational efficiency without compromising plan quality. Within the CyberKnife TPS, the “target boundary distance” option allows targeting of beams at a specific distance from the delineated target boundary. This parameter can be adjusted by the planner to emphasize either homogeneity or conformity. In this study, a target boundary distance within 5 mm outward was commonly employed.

ZAP-X and CyberKnife treatment planning systems share several design features. One notable feature is the ability to create multiple hollow contour sets, known as “shells”, which provide control over plan conformity and dose falloff. Planner can adjust the objectives and penalties assigned to these shells to fine-tune plan quality in inverse planning. In this study, the CyberKnife planer typically generated three sets of shells to manage spillages at different dose levels (e.g., 3 mm, 10 mm, and 20 mm from target boundary for high, middle, and low dose regions, respectively). The sizes of these shells were adjustable based on the planer’s preference. In contrast, the ZAP-X TPS offers predetermined shell sizes to planers, including 0 mm, 1 mm, 5 mm, and 10 mm from the target boundary. Another shared feature is that both systems support the ray-tracing algorithm in dose calculation for tissue heterogeneity, although CyberKnife TPS offers the additional option of Monte Carlo dose calculation. For this study, we employed the ray-tracing algorithm for dose calculation in both treatment planning systems.

In this study, the primary objective for both ZAP-X and CyberKnife plans was to achieve target-volume coverage between 97–100% while determining the prescribed isodose percentage (PIP) relative to the maximum dose. Additionally, stringent adherence to the dose constraints specified in the AAPM TG101 guidelines was maintained for all OARs (38). Special emphasis was placed on minimizing the extent of low dose regions to prevent any 30% isodose regions from extending beyond the immediate target vicinity.

### 2.4 Comparison metrics

Dose conformity of each plan was assessed using the Radiation Therapy Oncology Group (RTOG) conformity index (CI) and the modified Paddick conformity index (nCI) defined as follows:


(1) $$CI=\frac{V_{Rx}}{V_{T}}$$

(2) $$nCI=\frac{V_{T}\times V_{Rx}}{{\left(V_{T,\;Rx}\right)}^{2}}$$

Herein, $V_{T}$ is the planning target volume, $V_{Rx}$ is the total volume covered by prescription dose, and $V_{T,\;Rx}$ is the partial volume of target covered by prescription dose.

Dose homogeneity was assessed for each target, with the homogeneity index (HI) defined by the equation,


(3) $$HI=\frac{D_{max}}{D_{Rx}}$$

where $D_{max}$ is the maximum dose to the target and $D_{Rx}$ is the prescription dose.

The dose fall-off was assessed for each plan using the prescription isodose volume, i.e., $V_{x\%Rx}$ , which represents the volume receiving at least x% of prescription dose. Two commonly used gradient indices (GI) are calculated as follows:


(4) $$GI50\%=\frac{V_{50\%RX}}{V_{100\%Rx}}$$

(5) $$GI25\%=\frac{V_{25\%RX}}{V_{100\%Rx}}$$

In addition, two more surrogates were also calculated as follows,


(6) $$R50\%=\frac{V_{50\%RX}}{V_{T}}$$

(7) $$R25\%=\frac{V_{25\%RX}}{V_{T}}$$

The R50% and R25% indices are useful for evaluating dose fall-off performance while accounting for differences in conformity. These indices express the ratios of absolute irradiated volumes to the target volume, which allows for a more accurate assessment of dose fall-off without the influence of variations in conformity.

V12 and V10, which are the volumes of normal brain tissue receiving at least 12 Gy and 10 Gy, were reported for plan comparison as predictors of brain necrosis for single-fraction treatments (39).

The delivery efficiency was evaluated with estimated treatment time of each fraction from TPS and MU coefficient defined as the following,


(8) $$C\_MU=\frac{MU/Fx}{Rx\times V_{T}}$$

The estimated treatment time was calculated as the dry run time plus estimated patient setup and imaging time interval. For CyberKnife, the patient initial setup time was set as 5 minutes in TPS, and the imaging time interval is 1 minute. For ZAP-X, the patient initial setup time is also 5 minutes, and there is no imaging time interval needed because ZAP-X imaging tracking does not interrupt treatment delivery.

To assess the difference of indices and parameters between the ZAP-X and CyberKnife plans, the two tailed t-test was performed, and a P value less than 0.05 was considered to indicate statistical significance.

## 3 Results

**Table 3** provides a summary of the comparison for conformity, homogeneity, dose falloff and delivery efficiency of all plans and targets between ZAP-X and CyberKnife. The results indicate that the CyberKnife system demonstrated statistically significant better CI and nCI (see Equations (1) and (2)) compared to ZAP-X(p values shown in **Table 3**). However, there is no significant difference in target-volume coverage between the two modalities. On average, CyberKnife plans exhibited statistically significant smaller HI (see Equation (3)), while the ZAP-X plans showed a lower minimum dose, higher mean dose and higher maximum dose, indicating greater dose heterogeneity within the targets. Conversely, the ZAP-X plans demonstrated better dose falloff, as indicated by the GI50%, GI25%, R50% and R25% indices (see Equations (4–7)), which reflect the benefits in the medium and low dose regions. Additionally, the ZAP-X plans achieved significantly smaller irradiated volume of normal brain tissues, as evidenced by the V12 and V10 parameters, while the mean dose to whole brain remained comparable between the two modalities. In terms of delivery efficiency, there was no significant difference in the estimated treatment time between modalities, although ZAP-X plans generally required fewer MUs compared to CyberKnife plans.

**Table 3:** Comparison of dosimetry indices and parameters for plan quality and delivery efficiency.

		Cyberknife	ZAP-X	p *Value*
		*Median (range)*	*Median (range)*	
Conformity	CI	1.28 (1.10–1.76)	1.41 (1.19 - 1.86)	<0.001
	nCI	1.30 (1.10–1.76)	1.41 (1.20 - 1.88)	<0.001
	Coverage(%)	99.87 (97.22 -100)	99.64 (98.46–100)	0.90
Homogeneity	HI	1.19 (1.07 - 1.27)	1.39 (1.13 - 2.00)	< 0.0001
	Dmin (%D_Rx_)*	1.00 (0.92 -1.11)	0.95 (0.81 - 1.01)	< 0.0001
	Dmean (%D_Rx_)	1.11 (1.04 - 1.15)	1.23 (1.09 - 1.44)	< 0.0001
Dose falloff	GI50%	5.27 (3.39 - 8.98)	3.14 (2.70 - 3.81)	< 0.0001
	GI25%	16.53 (8.17 - 31.02)	11.72 (7.76 - 18.64)	< 0.0001
	R50%	6.86 (3.95 - 12.85)	4.53 (3.57 - 5.48)	< 0.0001
	R25%	20.04 (10.18 - 41.70)	16.49 (10.90 - 25.20)	< 0.001
Normal brain	V12 (cc) ^**^	4.34 (1.23 – 20.84)	1.84 (0.51 – 13.64)	< 0.01
	V10 (cc)	6.05 (1.73 – 29.46)	2.69 (0.71 – 21.17)	< 0.01
Whole brain	Dmean (cGy)	116 (28 - 484)	111 (24- 445)	0.93
Delivery efficiency	C_MU(MU/(cGy∙cc))***	2.75 (0.26 - 9.68)	1.84 (0.31–6.81)	< 0.001
	Tx Time/Fx (mins)	28 (17–51)	26 (16–65)	0.99

* The minimum and mean doses of target are presented as percentages of the prescription dose.** The V12 and V10 of Normal brain tissue were calculated and compared for 12 plans with single fraction, all other parameters were compared for 28 plans.*** The coefficient C_MU is defined in Equation (8).

**Figure 1:**
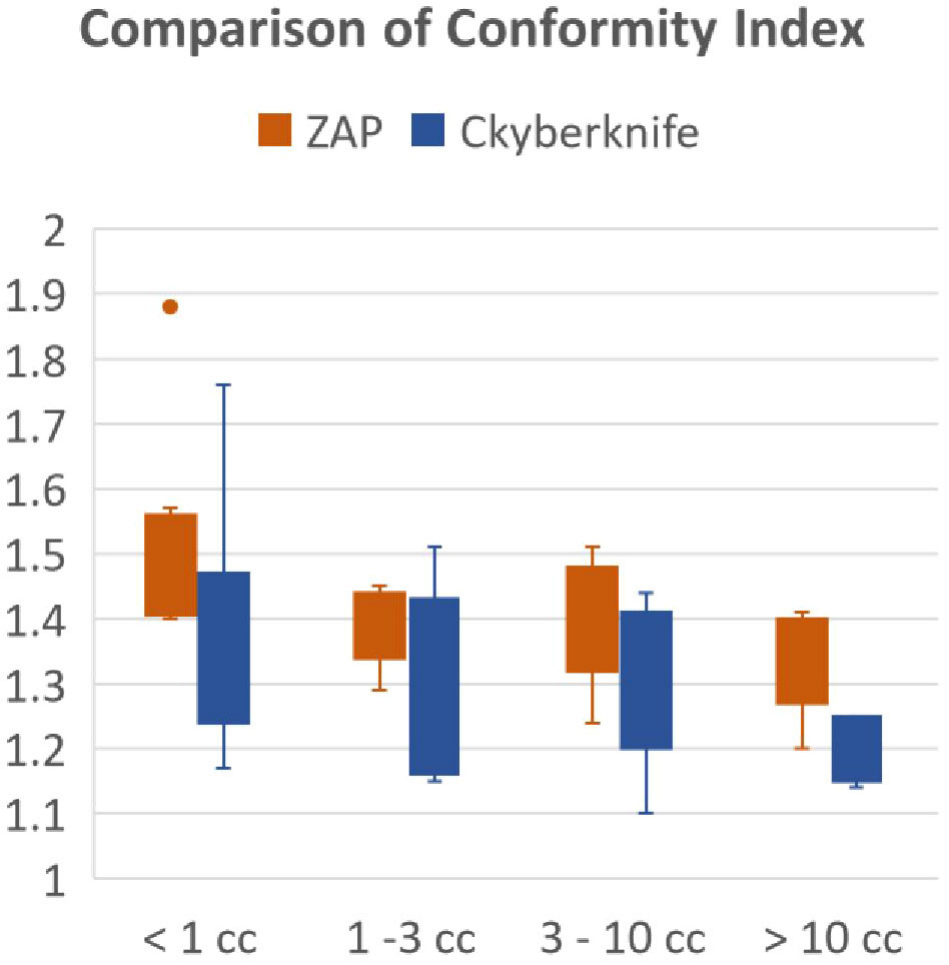
The results of the modified Paddick conformity index were compared using box plots, which were divided into four separate target size ranges.

The comparison of plan conformity was further refined based on target sizes. **Figure 1** illustrates the graphical comparison of CI for plans generated by ZAP-X and CyberKnife across various target size ranges. The overall findings indicate that CyberKnife plans exhibit superior conformity compared to ZAP-X plans. Particularly, for very small tumors (< 1 cc), CyberKnife achieved significantly smaller CI values than ZAP-X. Among the 9 plans evaluated, the mean CI values for ZAP-X and CyberKnife were 1.53 and 1.38, respectively. However, the difference in CI was less pronounced for intermediate-sized tumors. Among the 14 plans with medium-sized targets (between 1 cc and 10 cc), the mean CI values for ZAP-X and CyberKnife were 1.38 and 1.31, respectively. Conversely, for large tumors (> 10 cc), the disparity in CI became more significant. Among the 5 plans assessed, the mean CI values for ZAP-X and CyberKnife were 1.34 and 1.19, respectively.

**Figure 2:**
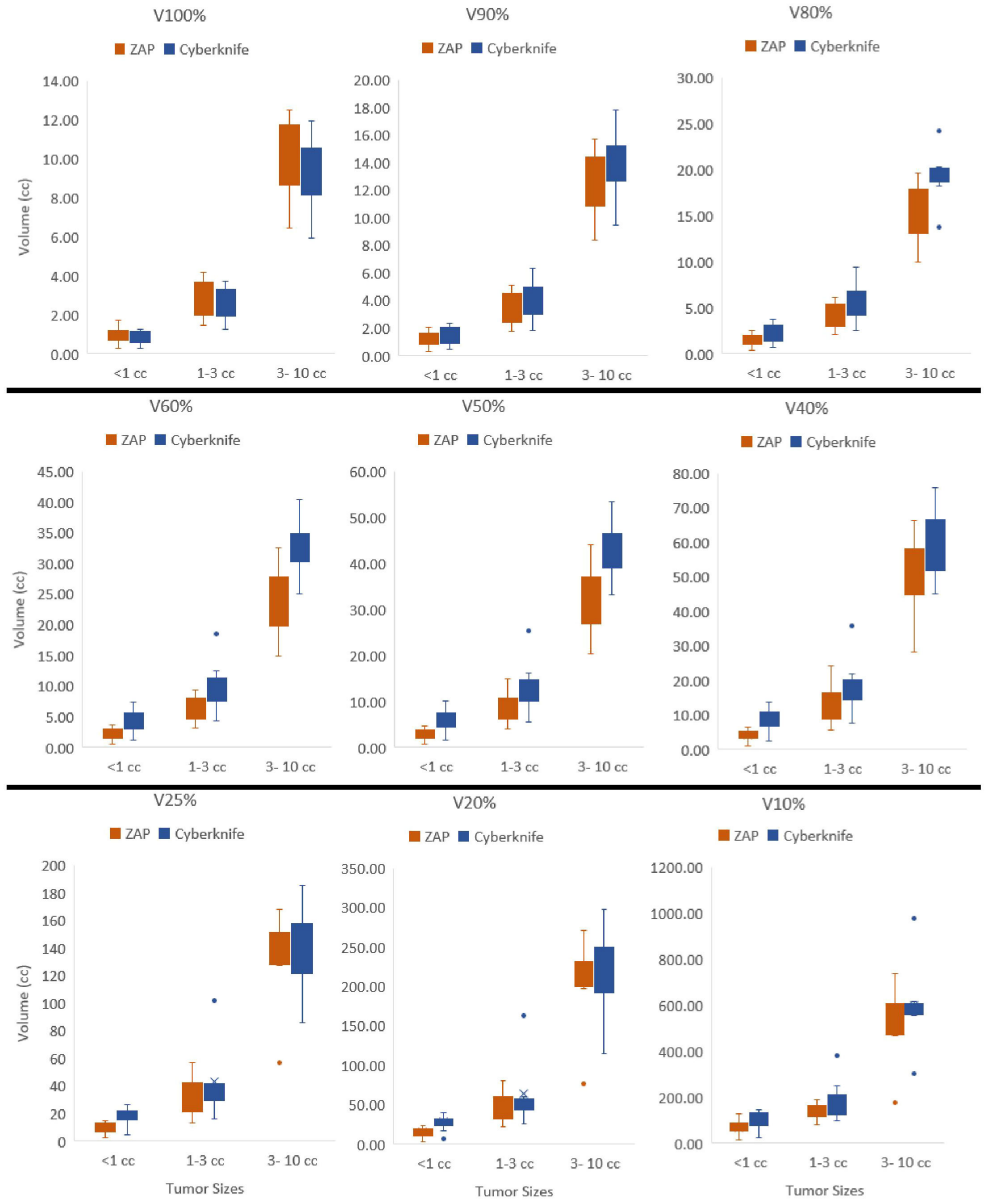
Isodose volumes were displayed as box plots comparing two modalities, i.e., V100%, V90%, V80%, V60%, V50%, V40%, V25%, V20% and V10% are the volumes enclosed by 100%, 90%, 80%, 60%, 50%, 40%, 25%, 20% and 10% of isodose surfaces normalized with prescription dose. The results were divided into three separate target size bins, i.e.,<1cc, 1–3cc and 3–10cc.

**Figure 2** presents a detailed comparison of isodose volumes at various percentage levels, ranging from 100% to 10%, with small increments. The analysis included 23 plans with target volumes less than 10 cc, with a median volume size of 1.70 cc, ranging from 0.15 cc to 8.67 cc. Across three target size ranges demonstrated in **Figure 2**, CyberKnife consistently outperformed ZAP-X in terms of V100%, demonstrating superior conformity. For targets smaller than 1 cc, ZAP-X exhibited better dose fall-off from V90% to V10% compared to CyberKnife. For targets larger than 1 cc but less than 10 cc, ZAP-X showed statistically significant superiority over CyberKnife from V90% to V40%. However, no noticeable differences were observed between the two modalities in the low-dose range (V25% to V10%) for targets between 1 cc and 10 cc. Analyzing the 14 plans with target volumes between 1 cc and 10 cc, the mean R25% values for ZAP-X and CyberKnife were 17.1 and 20.6, respectively, with a p-value of 0.096. Additionally, there were five plans with a target-volume range larger than 10 cc, not depicted in **Figure 2**, with a median volume size of 21.96 cc, ranging from 14.44 cc to 25.83 cc. Comparing the dose falloff performance between ZAP-X and CyberKnife for these five plans, the mean R50% values were 4.55 and 4.38, respectively, with a p-value of 0.66. The mean R25% values for the two modalities were 14.6 and 13.1, respectively, with a p-value of 0.33. These findings indicate that there are no observable differences in both the medium and low-dose regions between the two modalities for targets larger than 10 cc.

**Figure 3:**
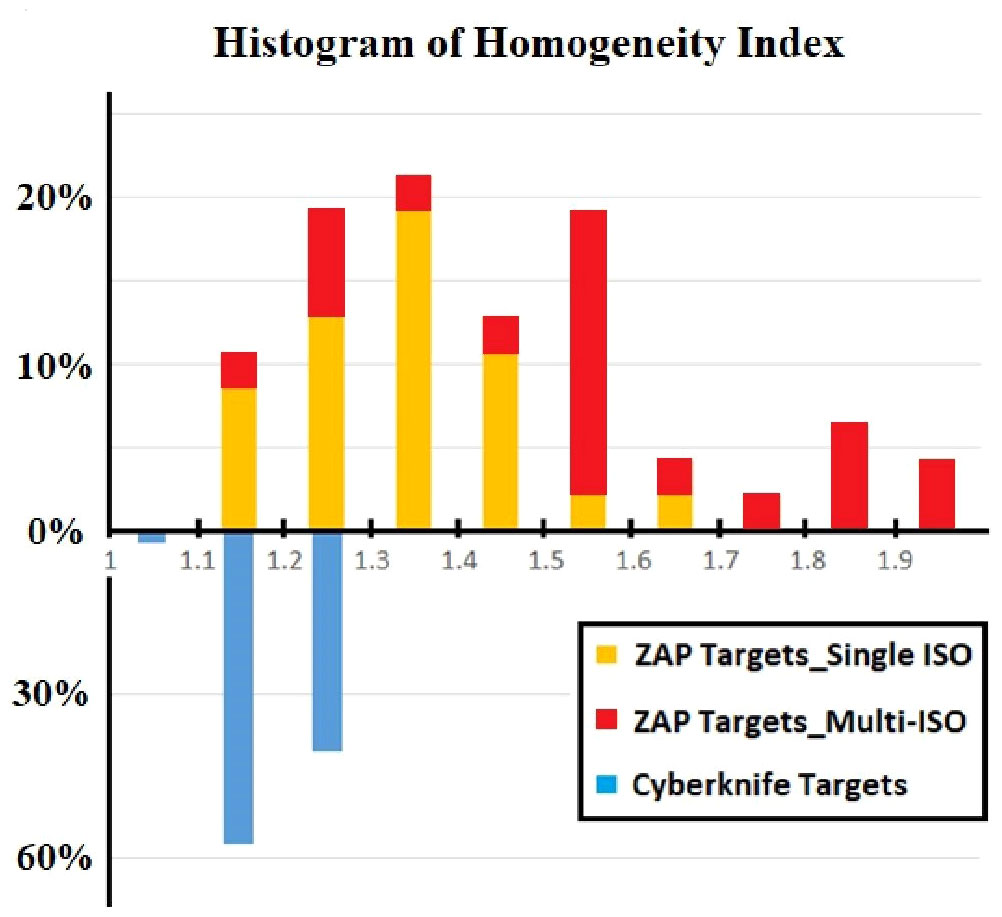
The comparison of HI was performed using differential histogram. The range of HI value is from 1.0 to 2.0 with bins of 0.1 for all 47 lesions. For each of bin of ZAP-X histogram, the targets covered by single and multiple isocenters were differentiated by different colors.

**Figure 3** displays a comparison of the HI results for all lesions treated with CyberKnife and ZAP-X. The results are presented as histograms, representing the percentage of targets within each HI range. In the CyberKnife plans, all targets achieved an HI value of less than 1.3. Conversely, the ZAP-X plans exhibited a broader distribution of HI values, with 83% of targets having an HI less than 1.6, and 64% of targets with an HI less than 1.5. Notably, the HI distributions differed between the ZAP-X targets treated with a single isocenter and those treated with multiple isocenters. Among all ZAP-X targets, 55.3% were covered by a single isocenter, with a median HI value of 1.31, and 92.3% of these targets achieved an HI less than 1.5. For the remaining 44.7% of targets treated with multiple isocenters, 71.4% of them exhibited an HI greater than 1.5.

**Figure 4:**
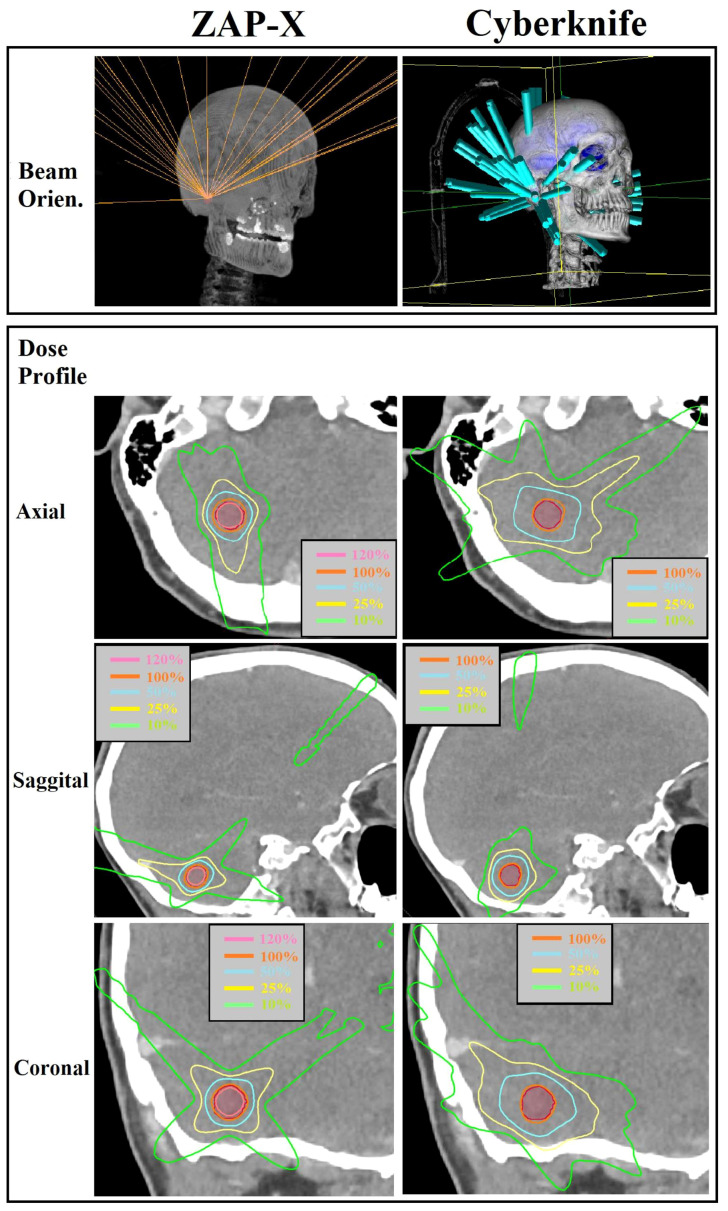
Visual comparison of beam orientation and multiplanar dose distributions between ZAP-X and CyberKnife for a representative case with small target volume (i.e., 0.84 cc). The 100% isodose line was normalized to prescribed dose.

In **Figure 4**, a comparative analysis of dose distributions for a small target in the skull base is presented. The optimized beam orientations of the two modalities are influenced by the target’s location, resulting in distinct dosimetric characteristics. The cut views of 2D dose distribution reveal that ZAP-X benefits from its posterior beams, which offer the shortest radiological paths to the target. On the other hand, CyberKnife relies on its lateral and superior beams. The ZAP-X plan utilized a single 12.5 mm collimator size isocenter, resulting in 99.3% target-volume coverage, a CI of 1.40, a HI of 1.45, and a GI50% of 2.81. In contrast, the CyberKnife plan employed collimation sizes of 10 and 12.5 mm, achieving 100% target-volume coverage, a CI of 1.48, an HI of 1.23, and a GI50% of 5.56. Additional plan parameters related to delivery efficiency can be found in **Table 4** under plan ID 5.

**Table 4:** Comparison of beam parameters and delivery efficiency of ZAP-X/Cyberknife for 18 plans with a single target.

Plan ID	Target Vol.(cc)	ZAP-X ISO No.	ZAP-X/Cyberknife^*^				
			Collimator Size Range(mm)^**^	Beam No.^***^	Tx Time (mins)		C_MU(MU/(cGy∙cc))
1	0.15	1	(7.5,7.5)/(10,10)	36/42(39)	18/19		20/18
2	0.42	1	(10,10)/(10,10)	35/90(40)	19/24		6.7/8.0
3	0.47	1	(10,10)/(10,10)	32/97(49)	16/20		6.8/9.1
4	0.48	1	(12.5,12.5)/(10,10)	33/32(24)	16/19		3.5/8.2
5	0.84	1	(12.5,12.5)/(10,12.5)	36/51(32)	18/17		3.6/5.8
6	0.89	5	(10,15)/(10,10)	51/114(39)	18/23		3.3/7.2
7	0.92	1	(15,15)/(10,10)	48/82(31)	16/27		2.7/5.5
8	1.1	3	(10,12.5)/(7.5,10)	70/100(49)	23/27		4.3/6.1
9	1.13	2	(10,12.5)/(10,10)	48/129(37)	21/28		2.7/4.0
10	2.76	1	(20,20)/(10,10)	33/200(57)	16/28		0.7/1.7
11	2.9	3	(10,25)/(10,10)	52/104(20)	22/31		1.5/2.6
12	4.86	4	(12.5,25)/(10,20)	34/104(45)	20/23		0.47/0.93
13	8.41	5	(15,25)/(10,20)	92/190(59)	32/26		0.60/0.69
14	8.67	10	(12.5,25)/(10,25)	100/114(46)	35/25		0.81/0.69
15	14.44	10	(15,25)/(10,20)	114/190(48)	36/34		0.43/0.60
16	21.96	12	(15,25)/(10,30)	197/192(66)	43/31		0.33/0.27
17	22.94	12	(20,25)/(10,30)	122/185(44)	40/32		0.31/0.33
18	25.83	9	(15,20)/(10,30)	190/180(46)	38/31		0.34/0.26

*All parameters are listed with the order ZAP-X/Cyberknife. ** The range is presented as the value of (min, max) *** The node number of each Cyberknife plan is in parenthesis.

**Figure 5:**
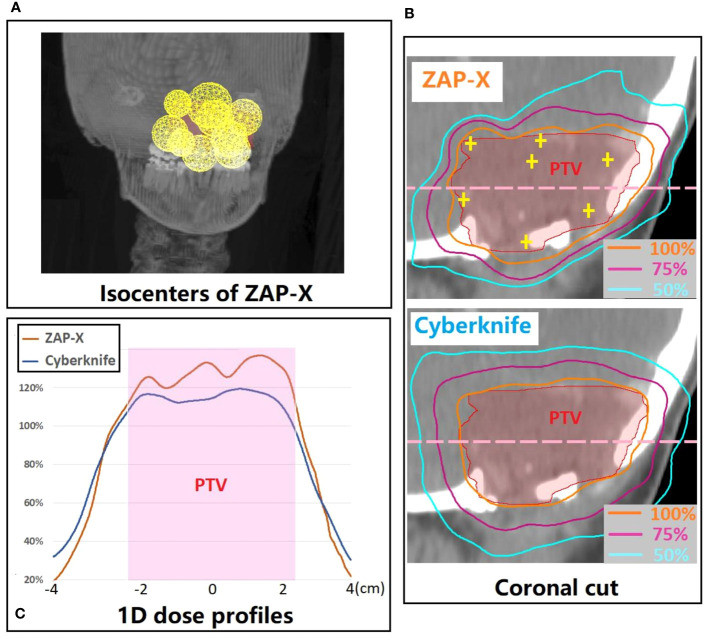
**(A)** 3D visualization of the isocenter positions in the ZAP-X plan for a representative postoperative case with large PTV (i.e., 21.96 cc). **(B)** A coronal cut view which demonstrates the difference in dose distribution between ZAP-X and Cyberknife. The yellow cross-hairs indicate the isocenter positions of ZAP-X plan projected at the view. **(C)** Comparison of 1D dose profiles between modalities along the pink dash line in **(B)**. The 100% isodose line was normalized to prescribed dose.

**Figure 5** presents a postoperative case with a large target volume. For ZAP-X, a total of 12 isocenters were used with collimation sizes ranging from 15 to 25 mm. The strategic placement of most isocenters near the boundary of the target volume aimed to achieve optimal plan uniformity. The resulting ZAP-X plan demonstrated excellent performance with 99.7% target-volume coverage, a CI of 1.19, a HI of 1.52, and a GI50% of 3.02. In comparison, the CyberKnife plan achieved 98.7% target-volume coverage, a CI of 1.14, an HI of 1.22, and a GI50% of 3.95. Further details on delivery efficiency and other plan parameters can be found in **Table 4** under plan ID 16.

**Table 4** provides a comparison of plan parameters for all plans targeting a single lesion within the cohort. The plans are listed in ascending order based on the sizes of the target volumes. Strong correlations were observed between the MU numbers of the plans with the target volume sizes, also, with the selected collimator sizes. In this study, most selected collimator sizes for both modalities were 10 mm or larger, with none smaller than 7.5 mm. CyberKnife plans consistently employed a minimum collimator size of 10 mm across all cases. Additionally, for target sizes less than 3 cc, a single collimator size of 10 mm was frequently utilized for CyberKnife plans. Conversely, the chosen minimal collimator size for ZAP-X plans varied based on the specific target size and shape. For very small lesions (plans with IDs 1 to 5 in **Table 4**), both modalities employed similar collimator size ranges. ZAP-X exhibited a slight improvement in delivery efficiency, requiring 15.8% fewer MUs and 2.4 minutes less in estimated delivery time, on average, compared to CyberKnife. However, these differences did not reach statistical significance (p=0.27 and 0.08, respectively). For lesions with intermediate sizes up to 3 cc (plans with IDs 6 to 11 in **Table 4**), CyberKnife plans utilized smaller maximal collimator sizes compared to ZAP-X. For these cases, ZAP-X demonstrated notable delivery efficiency, necessitating 44.3% fewer MUs and 8.2 minutes less in estimated delivery time, with statistically significant differences observed (p=0.007/0.002). In the cases of target sizes exceeding 3 cc (plans with IDs 12 to 18 in **Table 4**), the largest 25 mm collimator size of ZAP-X is frequently selected, while the maximum collimator size for CyberKnife plans reached up to 30 mm. ZAP-X exhibited a 12.6% reduction in MUs without statistical significance (p=0.40), but a statistically significant average increase of 6 minutes in estimated delivery time (p=0.02) compared to CyberKnife.

## 4 Discussions

This study presents a systematic comparison of treatment plans between ZAP-X and CyberKnife. The findings indicate that both modalities can generate treatment plans with acceptable plan quality, as shown in **Table 1**. In general (without considering the impact of target size), CyberKnife outperforms ZAP-X in terms of plan conformity and homogeneity, whereas ZAP-X demonstrates superior performance in dose gradient. The treatment delivery efficiency is comparable between the two modalities. Furthermore, the study reveals a correlation between dosimetry performance and delivery in relation to the target size, as illustrated in **Figures 1**, **2**, **Table 4**. Specifically, for small target sizes (<1 cc), CyberKnife exhibits significant advantages in plan conformity, while ZAP-X excels in dose gradient. However, for larger target sizes, the respective advantages become less apparent.

CyberKnife employs non-isocentric beam delivery, utilizing gantry pivoting with a robotic arm to enhance flexibility. This unique feature enables the generation of highly conformal and uniform treatment plans, as evidenced by **Figures 1**, **3**, particularly for cases involving irregular or large volumes, as illustrated in **Figure 5**. The CyberKnife TPS supports the creation of plans with strategically directed non-isocentric beams near the target’s boundary. This approach ensures that the prescribed isodose line conforms to the target volume’s surface curvature, while minimizing beam overlap within the target, thereby enhancing plan homogeneity (40), as demonstrated in **Figures 5B, C**.

In contrast, ZAP-X utilizes an isocentric technique that lacks the gantry pivoting flexibility seen in CyberKnife. Instead, it relies on sphere packing, similar to Gamma Knife, to address complex cases, taking advantages of its beam geometry with large solid angles. Notably, ZAP-X exhibits greater capability than Gamma Knife in manipulating shot shape by delivering optimized sparsely distributed beams to an isocenter. In cases involving multiple isocenters, once the isocenters with appropriate collimator sizes are positioned, all candidate beams associated with the isocenters are simultaneously optimized. The planning process typically involves iterative adjustments, such as manual fine-tuning of isocenter positions and collimator sizes combined with inverse planning guided by updated dose constraints. While the planner can steer the optimizer by adjusting the dose constraints, the determination of isocenter positions and collimator sizes is crucial for achieving the desired plan quality in many situations.

Generally, if multiple isocenters are placed more peripherally on the target, the resulting plan tends to exhibit improved homogeneity, as demonstrated in **Figure 5B**. However, due to the overlap of neighboring shots, hotspots may be unavoidable, as depicted in **Figure 5C**. Planners prioritize target coverage and conformity while allowing for compromises in plan homogeneity, which explains for the variation in HI values of ZAP-X plans with multiple isocenters, ranging from 1.1 to 2.0, as depicted in **Figure 3**.

In **Figure 3**, we also present an interesting finding regarding the comparison of HI-value distributions between ZAP-X plans of single isocenter and plans with multiple isocenters. It is observed that plans utilizing a single isocenter have yielded better HI values compared to plans with multiple isocenters. The median HI value of the single isocenter plans is close to that of CyberKnife. As depicted in **Figure 4**, in this study, a commonly employed strategy in ZAP-X planning is to use a single isocenter with a collimator size that closely matches the target diameter. This approach often leads to achieving an acceptable CI, particularly when dealing with regular target shapes. In the plans with a single isocenter, the HI value is determined by the prescribed isodose line based on desired target coverage and plan conformity, which is influenced by the flatness, shape and dose falloff of shot with optimized beams.

Although ZAP-X and CyberKnife both possess large number of spatial nodes covering solid angles for radiation crossfire exceeding 2π, their respective available scopes of beam orientations differ significantly, i.e., the scope of beam orientation of ZAP-X are quite similar to Gamma Knife, whereas CyberKnife possesses the scope covering entire anterior hemisphere, however, posterior beams are restricted in access due to the limited conch height. As depicted in **Figure 4**, these difference in beam orientations may lead to different characteristics in dosimetry, and such difference may be influenced by the anatomical location of target.

Although ZAP-X and CyberKnife both utilize a large number of spatial nodes to cover solid angles exceeding 2π steradians for radiation crossfire, they differ in terms of their available scopes of beam orientations. ZAP-X exhibits a beam orientation scope similar to that of Gamma Knife, while CyberKnife has a scope nearly covering the entire anterior hemisphere. However, CyberKnife’s access to posterior beams is limited due to the restricted couch height. This difference in beam orientations can result in discernible differences in dosimetric characteristics as depicted in **Figure 4**, and ultimately lead to variations in plan quality, particularly when considering the specific anatomical location of the target.

**Tables 1**, **4** provide evidence that, overall, ZAP-X demonstrates superior delivery efficiency in terms of MU numbers compared to CyberKnife within the studied cohort. It is important to consider that several factors can influence the MU numbers of plans using different modalities. For example, as indicated in **Table 5**, ZAP-X has a higher output per MU than CyberKnife at the same collimator size, while CyberKnife beams possess better penetrability, evidenced by the comparison of TPRs. However, as summarized in **Table 4**, the key determinant affecting the MU numbers among plans is often the selection of different collimation size ranges during plan creation. For plans with intermediate target sizes (i.e., plans with ID 6–11 in **Table 4**), CyberKnife planners, drawing from their experience, typically avoid selecting collimators larger than 10 mm. Conversely, for larger target volumes, CyberKnife benefits from the availability of larger collimator sizes that are not available on ZAP-X. On average, across the entire cohort, no significant difference in estimated treatment time is observed between the two systems, as the movement time of the gantry dominates the total delivery time for both modalities. Consequently, small variations in MU numbers are unlikely to result in discernable differences in delivery time.

**Table 5:** Comparison of output and TPR@5cm for each collimator size using in planning.

Collimator Size(mm)	ZAP/Cyberknife^*^	
	Output (cGy/MU)	TPR@5 cm %
30	NA/0.98	NA/89.6
25	1.00/0.97	79.0/88.1
20	0.99/0.96	78.1/87.3
15	0.98/0.94	77.1/86.1
12.5	0.97/0.92	76.5/85.6
10	0.95/0.89	75.8/84.4
7.5	0.92/0.80	75.3/83.3

*Parameters are listed with the order ZAP-X/Cyberknife; outputs of IRIS^TM^ collimation are listed for Cyberknife; machine output of Cyberknife was calibrated with 60 mm fixed cone.

It is important to note that this study focuses solely on the systems with circular collimation and does not include the MLC version of CyberKnife. Additionally, it should be emphasized that the dosimetric characteristics highlighted in this study do not directly reflect the machine characteristics of the two systems, but rather the differences in dosimetric performance between the two modalities within the specific clinical implementation. These differences are influenced by machine characteristics, preferred clinical goals, and the current treatment planning capacities. The planning strategy employed in our study for both ZAP-X and CyberKnife prioritized target coverage, conformity and sparing of OARs. For plan homogeneity, a prescription isodose percentage (PIP) higher than 75% was desirable for CyberKnife plans, while PIP was maximized for ZAP-X without compromising conformity. Efforts were also made to balance dose falloff, treatment time, and other factors. Under the context of such clinical implementation, the extent of advantages of CyberKnife/ZAP-X plans in terms of plan homogeneity/dose falloff may be determined by the PIP chosen for planning and should not be concluded as a machine characteristic. Lee et al. (41) suggested that choosing a lower PIP can improve the dose falloff of CyberKnife plans while maintaining the desired plan conformity. As widely recognized in the radiosurgery community, the Gamma-knife employs a lower prescription isodose percentage, typically set at 50%, compared to the CyberKnife, which often uses 75% or higher. This is due to the machine characteristics and sphere packing technique of the Gamma-knife. In this study, it was demonstrated that ZAP-X, in some situations, can produce plans that approach the homogeneity achieved by CyberKnife, unlike the Gamma-knife.

The CyberKnife treatment planning system has undergone significant evolution and improvement over the last twenty years, transitioning from the initial forward planning scheme with isocentric sphere packing technique to the current, more sophisticated inverse planning scheme with non-isocentric technique. On the other hand, ZAP-X utilizes sphere packing to achieve conformal coverage of the target, and the dependence on planner experience remains a limitation of the current planning capacities for ZAP-X. The introduction of a more sophisticated optimization algorithm that allows for automatic placement of isocenters with proper collimator sizes based on the shape and size of the target is likely to further enhance plan quality.

The potential for enhancing plan quality with ZAP-X extends beyond algorithmic advancements alone. While intuitively increasing the number of isocenters in ZAP-X plans could potentially improve plan quality, practical limitations must be considered. Currently, skull position verification using kV imaging is required for each isocenter, which can significantly increase delivery time when more isocenters are added. In this study, the goal was to strike a balance between plan quality and treatment time by minimizing the number of isocenters in ZAP-X plans. However, it is anticipated that advancements in machine characteristics, such as increased gantry speed, improved imaging processes for skull alignment between isocenters, and the ability to dynamically adjust collimation during each isocenter delivery, have the potential to enhance delivery efficiency (42). These improvements, along with sophisticated planning schemes, hold the promise of achieving improved conformity and homogeneity in ZAP-X plans, bringing them closer to the levels achieved by CyberKnife.

In addition to machine characteristics that affect delivery behavior, the beam characteristics of different modalities can also have an impact on plan quality. As aforementioned, ZAP-X differentiates itself from CyberKnife with its lower beam energy, which is similar to Gamma Knife. Additionally, when considering a single beam, ZAP-X has a short SAD, which theoretically results in larger beam divergence and a sharper beam penumbra. These unique features have the potential to influence dosimetric performance, particularly in terms of dose falloff and peripheral dose. Further studies are needed to thoroughly investigate the dosimetric impact of these specific beam characteristics on plan quality.

This study has limitations regarding the inclusion of cases involving multiple lesions planned in a single treatment plan. As depicted in **Table 2**, only three plans were generated for treating more than three lesions simultaneously. It is crucial to acknowledge that CyberKnife provides IRIS collimator sizes up to 60 mm, which holds the potential for improved delivery efficiency and plan quality in cases with multiple lesions. Further research is warranted to comprehensively compare the performance of these two modalities in treating multiple lesions within a single plan.

## 5 Conclusions

Our study demonstrates that both ZAP-X and CyberKnife with circular collimation are capable of generating plans with equivalent dosimetric outcomes for patients with brain metastases of various sizes. Both modalities effectively achieve adequate dose coverage for the PTVs. While CyberKnife plans generally exhibit greater conformity and homogeneity, ZAP-X plans demonstrate a faster dose falloff. In terms of delivery efficiency, ZAP-X outperforms CyberKnife in terms of MU numbers, whereas the estimated delivery times of both systems are comparable. It is important to consider that the planning strategy employed for CyberKnife in this study was specific to a particular clinical implementation, utilizing a high PIP. Additionally, it is worth acknowledging that ZAP-X represents the first generation of the SRS platform, and its current planning and delivery scheme may have inherent limitations. However, as the technology continues to evolve and improve, it is anticipated that ZAP-X will unlock its full potential and deliver even better dosimetric performance.

## Data availability statement

The original contributions presented in the study are included in the article/supplementary material, Further inquiries can be directed to the corresponding author.

## Author contributions

YN: Writing – review & editing, Writing – original draft, Visualization, Validation, Resources, Project administration, Methodology, Investigation, Formal analysis, Data curation, Conceptualization. AR: Writing – review & editing, Methodology, Investigation, Data curation, Conceptualization. JL: Writing – review & editing, Methodology, Investigation, Data curation, Conceptualization. MC: Writing – review & editing, Conceptualization. DC: Writing – review & editing, Conceptualization. BC: Writing – review & editing, Conceptualization. AS: Writing – review & editing, Conceptualization. KU: Writing – review & editing, Conceptualization. DP: Writing – review & editing, Supervision, Resources, Project administration, Methodology, Investigation, Conceptualization.
